# Radiation Dose Evaluation in Pediatric Patients Undergoing Repeated Brain Computed Tomography Examinations

**DOI:** 10.3390/diagnostics16091265

**Published:** 2026-04-23

**Authors:** Mohammad Aljamal, Noor Abuasbi, Awadia Gareeballah, Zuhal Y. Hamd, Mohammed Alharbi, Amna M. Ahmed, Lama Almudaimeegh, Areej Hamami

**Affiliations:** 1 Department of Medical Imaging, Faculty of Allied Medical Sciences, Arab American University, 13 Zababdeh, Jenin P.O. Box 240, Palestine; nour.jamal1@icloud.com; 2 Department of Diagnostic Radiology, College of Applied Medical Sciences, Taibah University, Madinah 42353, Saudi Arabia; agsali@taibahu.edu.sa; 3 Department of Radiological Sciences, College of Health and Rehabilitation Sciences, Princess Nourah bint Abdulrahman University, P.O. Box 84428, Riyadh 11671, Saudi Arabia; zyhamd@pnu.edu.sa; 4 Medical Imaging Department, King Abdullah bin Abdulaziz University Hospital, P.O. Box 47330, Riyadh 11552, Saudi Arabia; moalharbi@kaauh.edu.sa; 5 Department of Radiological Sciences, College of Applied Medical Sciences, King Khalid University, P.O. Box 960, Abha 61421, Saudi Arabia; amustafa@kku.edu.sa; 6 Internal Medicine Department, College of Medicine, Princess Nourah bint Abdulrahman University, P.O. Box 84428, Riyadh 11671, Saudi Arabia; lmalmudaimeegh@pnu.edu.sa; 7 Department of Medical Imaging, Rafidia Surgical Hospital, Rafidia St., Nablus 9992200, Palestine; areej.hamami98@gmail.com

**Keywords:** pediatric brain CT, repeated scans, radiation dose, effective dose, CTDIvol, DLP, protocol optimization, ALARA

## Abstract

**Background**: Repeated brain computed tomography (CT) scans in children may result in substantial cumulative radiation exposure, particularly in young children, who are more sensitive to ionizing radiation. The purpose of the study was to assess the dose levels of radiation in patients who receive repeated brain CT during childhood and adherence rates to pediatric imaging protocols. **Methods**: A retrospective cross-sectional study was conducted among 177 patients aged ≤5 years who underwent two or more brain CT examinations with a total of 514 CT examinations. The information was gathered through the hospital Picture Archiving and Communication System (PACS), which included patient demographics, scan parameters, and scanner-reported dose indicators such as volume-averaged computed tomography dose index (CTDIvol) and dose-length product (DLP). The effective dose (ED) was calculated and compared with estimated doses based on a nominal pediatric CT protocol. **Results**: The findings indicated a great variation in scan parameters, with CTDIvol values of 8.9 to 51.7 mGy and DLP values of 177 to 1310 mGy.cm. The number of repeated scans showed a great increase in the cumulative ED (*p* < 0.001). The median doses in patients below the age of one year were greater than those in older children. There was also a closer relation of scanner-reported doses to adult protocols, which suggests a lack of an optimized pediatric setting. **Conclusions**: Children under 5 who undergo repeated brain CT scans may face excessive radiation exposure. The matter is aggravated by the fact that scans are performed repeatedly without optimization of the dose, which leads to significant cumulative ED.

## 1. Introduction

Computer Tomography (CT) has transformed medical diagnostics, providing speed and detail in image development unmatched before. It is especially important in the measurement of neurological emergencies in pediatric medicine, including congenital abnormalities [1,2]. Modern CT scanners have a high spatial resolution and short acquisition time; hence, the modality is the one of choice in emergency cases where timely diagnosis and life-saving is critical [3]. Nonetheless, there is a documented risk associated with this diagnostic power exposure to ionizing radiation. In comparison to the majority of other imaging modalities, CT presents a relatively high amount of radiation to the patient. Biological effects of this radiation, especially the stochastic effects (carcinogenesis and genetic damage), are currently of concern and study [4]. Linear No-Threshold (LNT), which is the model followed by most international protection agencies, assumes that radiation has no safe dose and that the risk of cancer is directly proportional to the dose, even at low levels. This principle helps to highlight the role of reducing all unnecessary exposure [5].

The issue is highly exaggerated among pediatric patients. There are various reasons why children are believed to be more radiosensitive than adults: their cells divide faster, their tissues are developing, and they have more remaining lifespan in which a radiation-related cancer can occur [6]. Evidence of a definite dose–response relationship has also been presented in epidemiological studies, including the study by Wang et al. (2023), which reports that cumulative radiation dose in childhood due to CT scans is associated with the risk of leukemia and brain tumors later in life [7].

The given risk is especially crucial in the case of a certain subpopulation of pediatric patients: patients with chronic neurological conditions who require several CT scans to be initially diagnosed, to plan their treatment, and for regular monitoring. Conditions such as hydrocephalus (that requires follow-up with shunts) [8], brain tumors, and severe traumatic brain injury may result in many scans within a short period [9]. These repeated examinations may result in a significant cumulative radiation dose, which may reach the level where the risk of cancer caused by the examination is a major clinical issue.

Repeated imaging procedures (CT and other forms of radiation) in a group of children less than the age of five led to high cumulative radiation exposure. This work approximated a significantly higher lifetime cancer risk (such as 7.6% in the leading group of the female population), which highlights the fact that very young children who experience frequent imaging are particularly susceptible [10]. In another control study, children with four or more CTs (particularly those having scans at or before the age of six) were found to be at high risk of leukemia, intracranial tumors, and lymphomas in comparison with unexposed children. This is an indication that frequent exposure to CT during early childhood significantly increases the risks of cancer [7].

From another perspective, a fundamental tool for optimization of CT radiation dose is the use of age-, size-, and indication-specific scanning protocols. Pediatric bodies are not just smaller counterparts of adult bodies, they have dissimilar tissue densities and attenuation qualities. The application of adult protocols to children will always lead to a high dose of radiation [11]. Global initiatives, such as Image Gently campaigns, have also helped in establishing and advancing the use of customized pediatric guidelines [12]. Additionally, Diagnostic Reference Levels (DRLs), which are commonly determined at the 75th percentile of dose distributions as per practice survey, are used to compare and identify areas of unusually high dosing practice and address them [13]. Thus, using adult protocols on pediatric patients exposes them to more radiation.

The fastest rate of brain development in children occurs in the first 2 years, and this reaches to 80% of the adult volume [14]. Thus, fetuses and young children (infants and toddlers) are the most vulnerable age groups to the radiation risk, as they are far more vulnerable to the long-term health consequences of radiation, especially cancerous occurrences [15,16].

Although the world has been made aware, it is still a major challenge in most parts of the world, especially in the Middle East. They have indicated a high level of failure to use pediatric-specific CT protocols, usually because of the lack of established national DRLs, inadequate training, or the use of scanner default settings [17,18,19]. Although other studies have investigated the use of CT dose in adults and justification practices [20,21], and the risk of CT radiation in children is well defined, there is still a lack of real-life information that can assess the variation in dose and compliance with the pediatric guidelines in repeated CT scans of the brain, especially in children below 5 years old in this area. The proposed study intends to fill this gap with specific evidence of clinical practices in the context and the possible opportunities to optimize them. Hence, the currently carried out study attempted to assess the effect of radiation dose CTDIvol, DLP, and ED on fetuses and young children who underwent repeated brain CT scans; compare the total ED of all CT scans performed by the scanner-administered dose with the nominal ED and the total nominal ED of a standardized pediatric protocol to determine compliance with best practices; and evaluate the changes in scan parameters and cumulative dose across repeated CT scans.

## 2. Materials and Methods

### 2.1. Study Design and Population

The study design was of a retrospective, cross-sectional type. The study population was pediatric patients with brain CT examinations on a Philips CT128-slice (Philips, Amsterdam, The Netherlands) scanner at the referral hospital in the city, which serves a population of approximately 100,000 people in the period between January 2023 and April 2025. Since radiation sensitivity is age-dependent, all the CT images acquired during the given period were examined. It was also discovered that a high percentage of the children studied were below 5 years old; hence, it was studied in this age group, since people are presumed to be the most sensitive to radiation during early life. The inclusion criteria included patients between newborns and 5 years old at the time of the initial scan, as well as those who had two or more CT scans of the brain. Patients who had only one scan or whose CT scans had artifacts in the image, or whose dose administration records were incomplete, were eliminated. The data were gathered through the review of the Picture Archiving and Communication System (PACS) of the hospital. The final analysis of the 245 patients included 177 pediatric patients who fit the criteria. The data were systematically collected in a standardized format to extract the following information for each eligible CT examination: patient demographics (age, gender); technical parameters (tube voltage (kVp) and tube current–time product (mAs); scanner-reported CT dose indices (CTDIvol and DLP); number of scans; and scan length (cm). Due to the retrospective nature of the study and reliance on PACS records, detailed clinical indications for each CT examination were not consistently available and, therefore, were not included in the analysis. The study was conducted at the main hospital, which provides a representative overview of routine clinical practice in this setting. The Arab American University committees gave ethical approval to this study (IRB number: R-2024/A/113/N).

### 2.2. Definition of Reference Protocols

After reviewing the imaging protocols for each patient, we observed variations in the protocols used, even for the same patient. Consequently, we retrieved the CTDI and DLP from the CT scan report for each patient. The ED was calculated by multiplying the DLP value by the age-specific conversion factor (k) for the head region, adopted from the established literature for children based on their age [22]. It should be noted that ED was estimated using age-specific conversion factors rather than patient-specific dosimetry methods such as size-specific dose estimates (SSDEs), which may introduce some degree of uncertainty in dose estimation. The total ED was obtained from all repeated CT scans for each patient individually. The nominal CTDI, DLP, and ED were also calculated using an optimized CT protocol tailored to each patient’s age category. This was done to confirm whether the total ED obtained from the CT scan report will be lower or higher than the ED if the scan were to be repeated using the nominal pediatric CT protocol. The reference available protocol provided by the vendor for this comparison used parameters of 100 kVp, 157 mAs, and a pitch of 1.5.

### 2.3. Statistical Analysis

The data were analyzed using SPSS version 27 (IBM, Armonk, NY, USA) and the numiqo online Statistics Calculator (numiqo Team, Austria 2025). Frequencies and percentages were used for demographic data, and inferential statistics were used with an average, median, and standard deviation. A Shapiro test was conducted to assess for normality of dose values (nominal ED, total nominal ED, total ED), demonstrating that the data was skewed as it contained clinically relevant outliers (Shapiro test *p* value less than 0.05); then, Spearman’s rho correlation, the Mann–Whitney U test, and the Kruskal–Wallis H test were used to assess the correlations between mean dose measurements and number of repeats, gender, and age groups, and a *p* value of <0.05 was considered statistically significant.

## 3. Results

**Figure 1:**
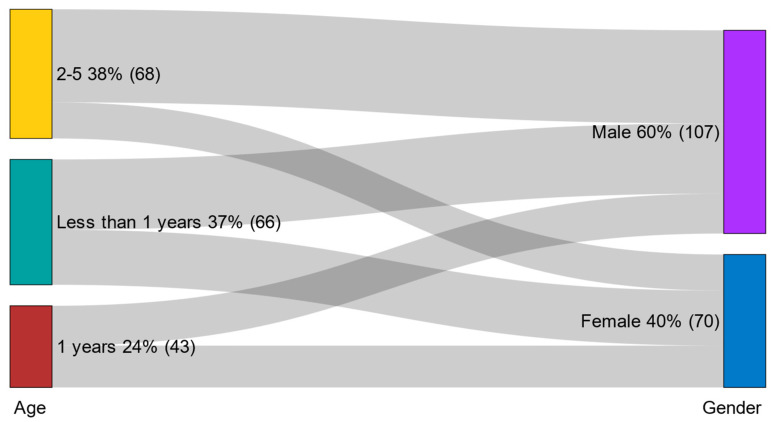
Frequency distribution of age and gender.

A total of 177 pediatric patients who underwent repeated brain CT scans were included in this study, with a total of 514 CT examinations. The age distribution showed that 37.3% of the patients were less than 1 year old, 24.3% were 1 year old, and 38.4% were between 2 and 5 years old. Most of the patients were male (60.5%), while females represented 39.5%. Regarding the number of repeated CT scans, the majority of patients (68.4%) underwent two scans, followed by three scans (13.0%) and four scans (7.9%). A smaller proportion of patients had five or more repeated scans (Table 1, Figure 1).

Table 2 presents the CT acquisition parameters and radiation dose indicators that were obtained in this study. The product of tube current and time was significantly different across the scans, with the results varying between 123 and 450 mAs, with a mean value of 277 ± 101. Tube voltage values were generally consistent between most examinations performed.

Indicators of radiation doses were also variable. CTDIvol was between 8.9 and 51.7 mGy, and DLP was between 177 and 1310 mGy.cm. This variation could be due to the difference in the size of the patient, the length of the scan, and the choice of the protocol used in the examinations.

The comparison of radiation dose with a difference between the two genders showed no statistically significant differences. The average ED was 6.90 mSv and 6.88 mSv in males and females, respectively (*p* = 0.417). Likewise, a nominal ED and the total nominal ED were marginally greater in females, but not statistically significant (*p* > 0.05) (Table 3).

However, there was a considerable age group difference (Table 4). Less radiation was given to the older groups, whereas more radiation was given to patients who were less than one year old. The median total ED among patients below one year of age was 7.11 mSv, 4.19 mSv among one-year-old children, and 2.78 mSv among children between 2 and 5 years of age. The same was found with nominal ED and total nominal ED, and the differences were significant (*p* < 0.001).

Further analysis showed a gradual increase in total radiation dose with the increasing number of repeated scans. The mean total ED increased from 3.88 mSv in patients who underwent two scans to 31.86 mSv in those who had eight scans. A similar pattern was observed for total nominal ED, which increased from 4.11 mSv in patients with two scans to 18.83 mSv for seven scans (increased as the number of scans increased). These differences were statistically significant (*p* < 0.001) (Table 5).

**Figure 2:**
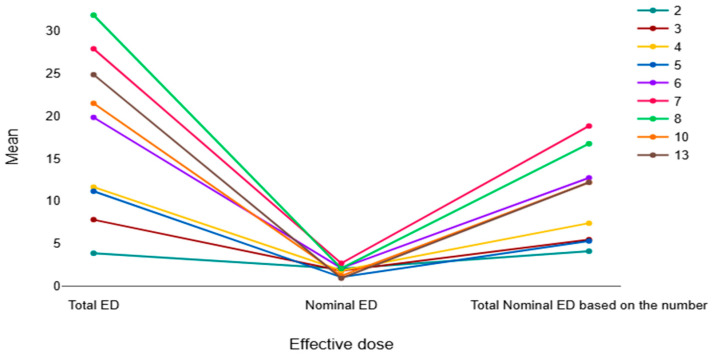
The graph shows the mean of total ED, nominal ED, and the total nominal ED based on the number of repeated CT scans.

In addition to the tabulated results, several figures were used to visualize the patterns of radiation dose with repeated CT scans. Figure 2 shows the trend in mean total ED, nominal ED, and total nominal ED according to the number of repeated CT scans. The figure clearly illustrates that the total ED and total nominal ED increased progressively as the number of scans increased, while the nominal ED per scan remained relatively stable.

**Figure 3:**
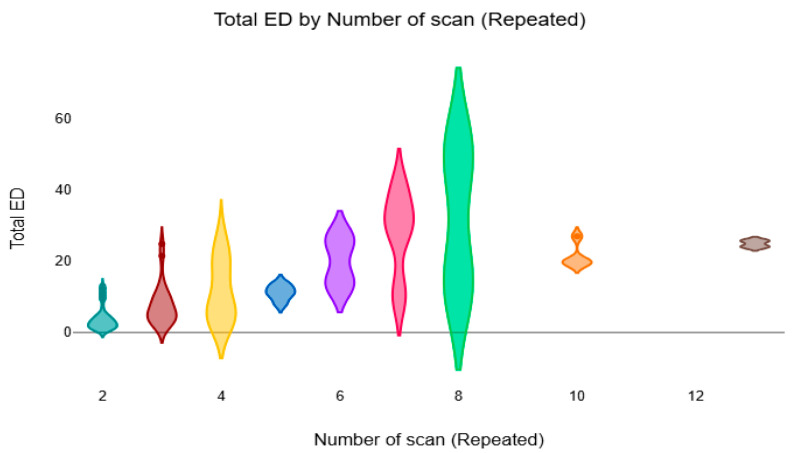
The violin plot compares the mean total ED based on the number of repeated CT scans.

Figure 3 presents a violin plot of the total ED across different numbers of repeated CT scans. The distribution evidently shows that the dose values increase with the increase in scan frequency, showing that the patients who had undergone scanning more than once had a higher cumulative radiation dose.

**Figure 4:**
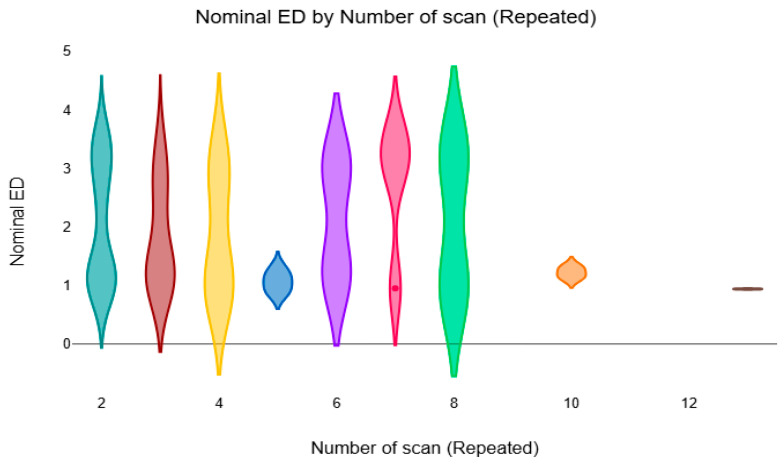
The violin plot compares the mean nominal ED based on the number of repeated CT scans.

Similarly, Figure 4 illustrates the distribution of nominal ED according to the number of repeated scans. In contrast to the total ED, the nominal ED values show less variation between groups, suggesting that the radiation dose per individual scan was relatively consistent despite the differences in the number of repeated examinations.

**Figure 5:**
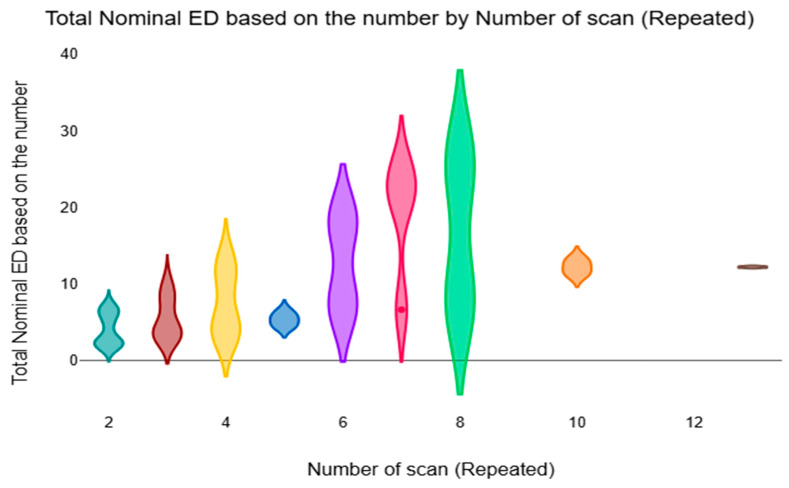
The violin plot compares the mean total nominal ED based on the number of repeated CT scans.

Figure 5 displays the distribution of the total nominal ED based on the number of repeated scans. The effect of repeated imaging was represented by the fact that the number of CT examinations was correlated with the total accumulated nominal ED.

**Figure 6:**
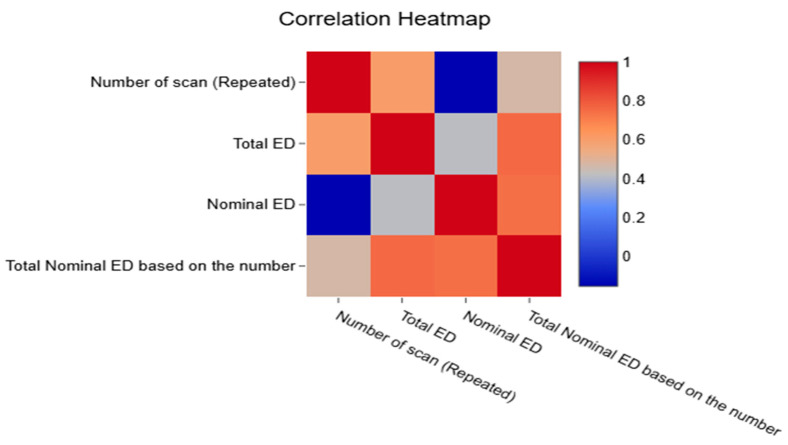
The correlation heatmap shows a strong association between the number of repeats and ED, and a moderate relationship between the number of repeats and total nominal ED based on the number of repeated CT scans.

The correlation heatmap demonstrates the relationships between the study variables (Figure 6). A strong positive association was observed between the number of repeated CT scans and the total ED, while a moderate relationship was found between the number of repeated scans and the total nominal ED based on the number of scans. These results reflect dose trends and variability; however, they do not account for the clinical justification of individual CT examinations.

## 4. Discussion

The current research has assessed radiation levels among children who had repeated brain CTs, with special consideration to children who are most sensitive to ionizing radiation. The results demonstrate that there are a number of significant concerns connected to radiation dose control and optimization in pediatric head imaging.

The findings of this study, among other important findings, are that the radiation doses administered in normal clinical practice were closer to those of adult CT protocols than those of pediatric protocols. This discrepancy indicates that scanning parameters might not be fully optimized to patient size and radiation sensitivity, although no clinical indication data is available to conclude about its appropriateness. Once the adult parameters are used on the children, the radiation dose may exceed that of diagnostic imaging by a significant margin. This is consistent with what has been reported in the previous research studying the practices of pediatric CT in different regions where non-adjustment of the protocol led to higher radiation dose levels [23,24].

The possible advantage of revising the protocol is evident in this study. It is observed that the ED decreased by 5.5–70.9% with compliance with an age-appropriate protocol, based on the comparison of the scanner-reported ED and the estimated dose calculated with the nominal pediatric protocol. This percentage is very high, especially among young children, especially if the patient has undergone repeated CT imaging. Children with neurological conditions, including hydrocephalus, traumatic brain injury, or brain tumors, tend to have multiple CT scans over time to make a diagnosis and for subsequent follow-up. In such cases, a small reduction per scan can result in a major decrease in the cumulative radiation dose during the early life of the patient. Therefore, this decreases the risk of cancer development in children, particularly at a young age. This is verified by previous research on the degree of the relationship between repeated CT imaging and the development of cancers [6,7]. These findings underscore the importance of carefully justifying CT examinations and optimizing radiation dose in pediatric imaging to minimize unnecessary exposure.

The other significant result of the study is the fact that there is a clear relationship between the cumulative radiation dose and the number of CT scans. The findings showed that the total ED was statistically significantly correlated with the increment in the number of repeated scans. It is a natural trend, yet it highlights the importance of careful clinical justification and optimization strategies when repeated CT imaging is required. Prior epidemiologic studies have indicated that cumulative radiation doses from CT during childhood could be linked to a higher risk of malignancies later in life, especially leukemia and brain tumors [6]. Consequently, one of the vital aspects of radiation protection in pediatric patients is the minimization of unwarranted repeated imaging.

The age-related differences in radiation exposure were also noted in this research. Children aged below one year were given a larger ED than other age groups. This observation could be due to a number of factors. Imaging may be done more frequently in younger children because such children have congenital or early neurological conditions that are likely to be monitored more closely. Also, technical reasons like scan length or imaging parameters are not necessarily improperly adjusted with very small patients. Considering the fact that the infancy period is one of the most radiosensitive periods of human development, these results once again emphasize the necessity of comprehensive adherence to the pediatric-specific imaging protocols. These findings are in line with another study conducted previously, showing that exposure to adult CT protocols accidentally done on children led to a significant rise in radiation dose levels. For example, the median CTDIvol of head CT was 23.9 mGy in the age-appropriate protocol and 37.5 mGy in the adult protocol [25]. However, in our study, the range of CTDIvol recorded for pediatric CT protocol patients was 8.9–12.6 mGy, while the adult CTDIvol could reach 51 mGy based on the CT imaging parameters used. Thus, this validates that scanning parameters showed significant variation in technical settings (tube current and dose indices) among examinations. Preferably, the imaging protocols are to be kept standard in size and clinical indication optimized. The presented variability, however, is an indication that the choice of protocols can be influenced by operator or scanner default settings instead of institutional protocol selection. Other studies have also found a great variation in CTDIvol and size-specific dose estimates across facilities practicing pediatric CT scans, which could be attributed to the variation in imaging protocols and operating practices [26]. These discrepancies may cause unnecessary dose differences among patients as well as among scans of one patient. The introduction of institutional guidelines and the periodic review of dose indicators would be a means to decrease this variability and achieve more consistent dose optimization. The lack of locally set Diagnostic Reference Levels (DRLs) in many regions, including the Middle East, could be a factor in the continued existence of non-optimized imaging practices. National or regional DRLs may thus be important for enhancing radiation safety in pediatric imaging [27]. Without a doubt, as well, educational campaigns and professional training programs have the potential to enhance knowledge of dose management in pediatrics and motivate more caution in the justification of repeat examinations. The importance of professional education in radiation protection has also been emphasized in recent research [28].

Previous studies have highlighted the significance of considering imaging techniques that are not based on ionizing radiation whenever they are clinically acceptable. Alternative non-ionizing imaging modalities that have been noted in the literature to be used in pediatric imaging include magnetic resonance imaging (MRI) and ultrasonography, among others, when clinically suitable [29]. Nevertheless, since these modalities were not measured in the current study, their contribution is mentioned here as a general consideration as opposed to a data-driven finding. It would be worthwhile to introduce modality comparison in future studies. Moreover, clinicians and radiologists should work closely on the selection of the most suitable imaging modality in each case, which will allow to reduce the amount of radiation exposure and achieve good diagnostic results.

Overall, the results of the present study can be added to the existing body of information, which highlights the necessity of better radiation dose management in pediatric CT scans. The identified gaps between the existing practice of clinical work and the suggested standards of pediatric imaging reveal the practical significance of this research in the areas where the optimization strategies may considerably improve patient safety and prove the influence of the selection of the correct protocols in the process of head CT imaging of a pediatric patient, specifically when it comes to repeated CT scan examinations.

There are a number of limitations in this research that should be taken into account in the interpretation of the results. First, we did the study in one medical center and used one CT scanner, which might not be generalizable to other institutions with other equipment and protocols. Second, the retrospective design was based on PACS data, and the detailed clinical reasons for repeated CT scans were not always available, which restricted the possibility of determining the suitability or the purpose of imaging. Thirdly, standard conversion factors were used to determine the effective dose as opposed to patient-specific dosimetry techniques like the size-specific dose estimates (SSDE), which could influence dose accuracy. Lastly, the research lacked clinical outcomes and long-term follow-up and could not directly determine health risks like cancer development. Despite these restrictions, the research gives great insight into radiation dose trends and outlines significant possibilities of enhancing the optimization of pediatric CT dose.

## 5. Conclusions

This study demonstrates that children under 5 years undergoing repeated brain CT examinations may experience substantial cumulative radiation exposure, with notable variability in dose parameters across scans. This issue is compounded once scans are re-done without an appropriate dose optimization, which may result in a large cumulative dose of radiation. The findings suggest potential gaps in protocol optimization in routine clinical practice. However, given the retrospective single-center design, the lack of clinical indication data, and the absence of patient-specific dosimetry and clinical outcomes, these results should be interpreted with caution. Future multicenter and prospective studies incorporating clinical justification and outcome assessment are recommended to better inform practice.

## Figures and Tables

**Table 1:** Demographic data and the number of repeated scans.

Demographic Characters		Frequency	Percent (%)
Age	<1 year	66	37.3
	1 years	43	24.3
	2–5	68	38.4
Gender	Male	107	60.5
	Female	70	39.5
Number of scans	2	121	68.4
	3	23	13.0
	4	14	7.9
	5	5	2.8
	6	2	1.1
	7	4	2.3
	8	2	1.1
	10	4	2.3
	13	2	1.1
Total		177 (514 scan)	100.0

**Table 2:** Distribution of CT scan parameters and radiation dose indicators.

Tube current–time (mAs)	Min.–Max.	123–450
	Mean ± SD.	277 ± 101
Tube voltage (kVp)	Min.–Max.	80–120
	Mean ± SD.	107 ± 10
CTDIvol (mGy)	Min.–Max.	8.9–51.7
	Mean ± SD.	28 ± 16.5
DLP (mGy.cm)	Min.–Max.	177–1310
	Mean ± SD.	624 ± 378

**Table 3:** Comparison of the total mean ED, nominal ED, and total nominal ED in both genders (regardless of the number of repeated scans).

ED		n	Mean	Median	Standard Deviation	*p* Value
Total ED	Male	107.00	6.90	3.76	6.86	0.417
	Female	70.00	6.88	3.76	9.01	
Nominal ED	Male	107.00	1.90	1.30	1.00	0.099
	Female	70.00	2.06	1.47	1.01	
Total Nominal ED	Male	107.00	5.33	4.45	3.95	0.548
	Female	70.00	5.59	4.94	4.31	

**Table 4:** Comparison of the mean total ED, nominal ED, and total nominal ED in different age groups (regardless of the number of repeated scans).

ED	Groups	n	Median	Mean Rank	Chi^2^	*p* Values
Total ED	Less than 1 year	66	7.11	113.08	26.96	<0.001
	1 years	43	4.19	86.49		
	2–5	68	2.78	67.22		
Nominal ED	Less than 1 year	66	3.21	144.17	133.15	<0.001
	1 years	43	1.3	76.6		
	2–5	68	0.97	43.29		
Total Nominal ED	Less than 1 year	66	6.66	134.27	85.07	<0.001
	1 years	43	2.88	72.5		
	2–5	68	2.23	55.49		

**Table 5:** Comparison of the mean total ED, nominal ED, and total nominal ED based on the number of repeated scans, and the percentage difference between total ED and nominal ED.

Number of Repeated Scans		Total ED	Nominal ED	Total Nominal ED	Percentage Difference Between the Mean Total ED and Total Nominal ED (%)
2	Mean ± Std. Dev	3.88 ± 3.27	2.056 ± 1.02	4.11 ± 2.05	5.5
	Median	2.841	1.462	2.92	
3	Mean ± Std. Dev	7.82 ± 5.85	1.83 ± 0.89	5.48 ± 2.68	35.3
	Median	5.972	1.433	4.298	
4	Mean ± Std. Dev	11.66 ± 8.42	1.85 ± 1.03	7.41 ± 5.48	44.5
	Median	7.07	1.37	5.48	
5	Mean ± Std. Dev	11.17 ± 2.19	1.06 ± 0.18	5.32 ± 0.89	70.9
	Median	11.172	1.089	5.446	
6	Mean ± Std. Dev	19.85 ± 8.48	2.12 ± 1.29	12.74 ± 7.72	43.6
	Median	19.848	2.124	12.743	
7	Mean ± Std. Dev	27.91 ± 12.6	2.69 ± 1.19	18.83 ± 8.30	38.9
	Median	30.445	3.108	21.756	
8	Mean ± Std. Dev	31.86 ± 25.2	2.09 ± 1.58	16.75 ± 12.64	62
	Median	31.861	2.094	16.754	
10	Mean ± Std. Dev	21.51 ± 3.66	1.22 ± 0.10	12.21 ± 0.99	55.1
	Median	19.907	1.217	12.166	
13	Mean ± Std. Dev	24.88 ± 1.17	0.94 ± 0.01	12.21 ± 0.12	68
	Median	24.876	0.939	12.208	
	*p* values Chi^2^	<0.001 68.13	0.145 12.14	<0.001 47.54	

## Data Availability

The original contributions presented in this study are included in the article. Further inquiries can be directed to the corresponding author.

## Abbreviations

The following abbreviations are used in this manuscript:

CT	Computed Tomography
LNT	Linear No-Threshold
ICRP	International Commission on Radiological Protection
DLP	Dose-Length Product
CTDIvol	CT Dose Index Volume
ED	Effective Dose
ALARA	As Low As Reasonably Achievable
PACS	Picture Archiving and Communication System
MRI	Magnetic Resonance Imaging
